# Transcriptomic analysis reveals sex-specific differences in the expression of *Dcl1* and *Fis1* genes in the radio-adaptive response of thymocytes to TRP53-mediated apoptosis

**DOI:** 10.1186/s12864-016-3036-0

**Published:** 2016-08-31

**Authors:** Pilar López-Nieva, Manuel Malavé, Laura González-Sánchez, José Fernández-Piqueras, Pablo Fernández-Navarro, Javier Santos

**Affiliations:** 1 Department of Cellular Biology and Immunology, Severo Ochoa Molecular Biology Center, Madrid Autonomous University (CBMSO-UAM), 28049 Madrid, Spain; 2 Institute of Health Research, Jiménez Díaz Foundation, 28040 Madrid, Spain; 3 Consortium for Biomedical Research in Rare Diseases (CIBERER), Madrid, Spain; 4 Carlos III Institute of Health, 28029 Madrid, Spain; 5 Cancer and Environmental Epidemiology Unit, National Center for Epidemiology, Carlos III Institute of Health, Madrid, 28029 Spain; 6 Consortium for Biomedical Research in Epidemiology and Public Health (CIBERESP), Madrid, Spain

**Keywords:** Radio-adaptive response, Thymocyte apoptosis, Caspase-3, cDNA microarrays, *Dlc1*, *Fis1*, Phosphorylation of TRP53 at serine 18 and serine 389, Sex differences

## Abstract

**Background:**

Radio-Adaptive Response (RAR) is a biological defense mechanism whereby exposure to low dose ionizing radiation (IR) mitigates the detrimental effects of high dose irradiation. RAR has been widely observed in vivo using as endpoint less induction of apoptosis. However, sex differences associated with RAR and variations between males and females on global gene expression influenced by RAR have not been still investigated. In addition, the response to radiation-induced apoptosis is associated with phosphorylation of TRP53 at both the serine 15 (ser-18 in the mouse) and serine 392 (ser-389 in mice) residues, but the role of these two phosphorylated forms in male and female RAR remains to be elucidated.

**Results:**

We analyzed the effect of administering priming low dose radiation (0.075 Gy of X-rays) prior to high dose radiation (1.75 Gy of γ-rays) on the level of caspase-3-mediated apoptosis and on global transcriptional expression in thymocytes of male and female mice. Here, we provide the first evidence of a differential sex effect of RAR on the reduction of thymocyte apoptosis with males showing lesser levels of caspase-3-mediated apoptosis than females. Analysis of transcriptomic profiles of 1944 genes involved in apoptosis signaling in radio-adapted thymocytes identified 17 transcripts exhibiting differential expression between both sexes. Among them, *Dlc1* and *Fis1* are closely related to the apoptosis mediated by the TRP53 protein. Our data demonstrate that overexpression of *Dlc1 and Fis1* occur concomitantly with a highest accumulation of phosphoserine-18-TRP53 and caspase-3 in radio-adapted thymocytes of female mice. In an opposite way, both down-modulation of *Fis1* and phosphoserine-389-TRP53 accumulation appear to be associated with protection from thymocyte apoptosis mediated by caspase-3 in males.

**Conclusions:**

Transcriptomic analysis performed in this work reveals for the first time sex-specific differences in gene expression influenced by RAR. Our results also suggest a sex-dependent dual role for phosphoserine-18-TRP53 and phosphoserine-389-TRP53 in the regulation of the radio-adaptive response in mouse thymocytes.

**Electronic supplementary material:**

The online version of this article (doi:10.1186/s12864-016-3036-0) contains supplementary material, which is available to authorized users.

## Background

The biological effects induced by exposing mammalian cells to ionizing radiation (IR) are closely associated with radiation doses and dose rates. Epidemiological evidence indicates that exposures of 0.2 to 3 Gy increase risk to cancer proportionally to the radiation dose received [1]. However, low dose radiation (<0.1 Gy) may result in a complex scenario of cellular responses that are either protective or supra-lethal according to the dose [2]. Radio-adaptive response (RAR) is a biological defense mechanism whereby a low dose of ionizing radiation (priming dose) protects cells against the detrimental effects of a subsequent higher radiation dose (challenging dose). This protective phenomenon was first demonstrated in vitro by Wolf and colleagues in 1984, who showed that human peripheral blood lymphocytes irradiated with tritiated thymidine had fewer chromosomal aberrations when they were subsequently irradiated with a high dose of X-rays [3]. RAR in human lymphocytes was later analyzed in vitro in a series of studies [reviewed in [4]]. Radioadaptation can be observed by a reduction of certain deleterious genetic effects related to DNA damage such as chromosomal aberrations, micronuclei formation, gene mutations, and DNA single- and double-strand breaks or by accelerated DNA repair [for review, see [5]]. In mammalian cells, induction of RAR requires a priming dose range of 0.01–0.2 Gy using low-LET (Linear Energy Transfer) radiation (X- or γ-rays) [6]. Furthermore, these cells need a time interval of 4–6 hours between the priming and the challenging doses to reach the full induction of radioresistance [7].

In vivo RAR has been investigated by whole-body X-ray exposure of male mice, using induction of apoptosis in splenocytes as the biological endpoint [8–10]. These studies demonstrated that reduction of apoptosis after the conditioning dose of X-rays may also be a hallmark of RAR. Additional investigations showed that less induction of apoptosis is closely associated with enhanced expression of the transformation related TRP53 protein in spleen cells of mice exposed to an acute single treatment with priming low dose irradiation prior to a subsequent high dose irradiation [11]. In contrast, primary cultures of mouse embryonic fibroblasts from *Trp53* knockout mouse were refractory to RAR in terms of decreasing the percentage of apoptotic cells [12]. Altogether, this evidence highlights a requirement of the *Trp53* gene in the induction of RAR.

It is well documented that DNA damage triggers a defensive cellular signaling response mediated by the *Trp53* gene [13]. Activation of human TRP53 protein in response to DNA damage induced by ionizing radiation occur concomitantly with phosphorylation at both the serine 15 and serine 392 (serine 18 and 389 in mouse TRP53) residues [14]. Phosphorylation of the human TRP53 protein at serine 15 (serine 18 in the murine TRP53) is a key event that rapidly occurs in response to radiation-induced DNA damage, thus contributing to its stabilization and functional activation as transcription factor by preventing MDM2 (a regulator of TRP53) from binding and by rendering TRP53 resistant to MDM2 [15]. For its part, modification of the phosphorylation site in TRP53 at serine 392 (homologue site of serine 389 in mouse TRP53) induces activation of site-specific DNA binding and tetramerisation, as prerequisites for its transcriptional activity [16, 17].

Cell type is one of the most important determinants of the *Trp53*-mediated cellular outcome [18], and the *Trp53* gene is required for radiation-induced apoptosis in mouse thymocytes [19]. A significant increase in the apoptosis index in thymocytes appeared after exposure of male mice to high or even low doses of whole-body γ-irradiation 6 h following the end of treatment [20]. In an opposite way, male mice receiving a low dose of 0.075 Gy priming X-irradiation administered 6 h prior to a high dose of 1–2 Gy of X-rays showed a reduction of apoptosis in thymocytes compared to males receiving high dose X-irradiation alone [21]. Still, there are no reports showing gender differences in apoptosis of radio-adapted thymocytes.

Transcriptional profiling is a sensitive way to study the effects of low dose radiation exposure [22, 23]. However, only one study has so far investigated global expression patterns after in vitro exposure to an adaptive regimen of radiation in three human lymphoblastoid cell lines, demonstrating down-modulation of apoptotic genes associated with RAR [24].

In this study we tested three hypotheses: (a) Exposure of thymocytes to acute low dose radiation (priming dose) prior to an acute high dose exposure (challenge dose) induces a differential apoptotic response in male and female mice; (b) sex-specific changes in the transcriptional expression of apoptosis-associated-genes induced by the priming dose occur in radio-adapted thymocytes; and (c) differences in apoptosis associated to RAR between sexes are related with variations in phosphorylation levels of TRP53 at serine 18 and serine 389.

## Methods

**Fig. 1:**
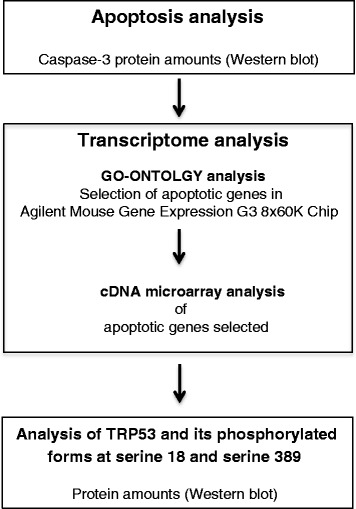
Signal cascade diagram showing working plan to test the proposed hypotheses

To test these hypotheses, several experimental approaches have been performed in thymocytes from male and female C57BL/6J mice (Fig. 1). First, caspase-3 amounts were estimated by Western blot to analyze the effect of administering priming low dose radiation prior to a high dose, as well as exposure to a single low or high dose of radiation, on the level of apoptosis. Second, we investigated by cDNA microarray analysis the transcript profiles of 1944 genes involved in apoptosis signaling (included in the Agilent Mouse Gene Expression G3 8x60K microarray) according to GO-Ontology database under the mentioned experimental conditions. Third, quantitative real-time reverse transcription polymerase chain reaction (RT-PCR) analysis was used to validate significant data from cDNA microarray analysis. Finally, protein amounts of phosphorylated forms of TRP53 at serine 18 and serine 389 were determined by Western blot in response to the adaptive regimen of radiation and after exposure to single low or high dose of radiation.

### Mice and irradiation

Male and female C57BL/6J mice were purchased from the Charles River Laboratories and kept 1 week in the local animal house for acclimatization. Animal experiments were carried out according to the European Commission Guidelines (Directive 86/609/CEE) on the use of laboratory animals.

Three groups of Irradiated males and females of comparable age (4 to 5 weeks) and weight (21–24 g) were used in this study: group subjected to an adaptive regimen of radiation (RAR group), low dose exposed group and group subjected to high dose exposure. Each experimental group consisted of three individuals. Mice from RAR group were irradiated with a priming dose of 0.075 Gy of X-rays, that it is known to known to induce an effective in vivo radiaodaptive response in mouse thymocytes [21], generated with a Philips MCN 101 X-ray generator operating at 100 kV/15 mA with 1 mm of Be and 3 mm AI added filtration. With an interval of 6 h, that it has been used for radio-adaptive studies with thymocytes [21], these mice were further irradiated with challenging dose of 1.75 Gy of γ-rays that were generated by a ^137^Cs gamma IBL-437C irradiator (CIS bio international, Gif-sur-Yvette, France). Mice from low dose irradiation group were exposed to a single dose of X-rays (0.075 Gy). Mice from high dose irradiation group were treated with 1.75 Gy of γ-rays alone. Control group consisted of non-irradiated mice.

### Thymic cell fractionation

Mouse thymus samples were mechanically dispersed and strained through a Nylon Mesh Cell Strainer of 40 μM (BD Biosciences, San Jose, CA) to isolate the thymocytes.

### Apoptosis analysis

Since pro-caspase 3 is cleaved into the 17–19 kDa and 12 kDa subunits only when cells undergo apoptosis, we used the fragmentation of caspase 3 as indicator of apoptosis induction. Cleaved caspase 3 (17/19 kDa) were determined by Western blotting and assessed by densitometry analysis.

### Transcriptome analysis

cDNA microarray experiments were performed using the Agilent Mouse Gene Expression G3 8x60K chip (Agilent Technologies, Palo Alto, CA). Three independent thymocyte samples for each experimental condition were used in this analysis. Total RNA was extracted by combination of Trizol reagent (Invitrogen, Carlsbad, CA), MaXtract high density extraction column (Qiagen, Hilden, Germany) and purification using the RNeasy Mini kit (Qiagen). Following extraction, total RNA were checked for RNA integrity (Agilent 2100 Bioanalyzer, Agilent Technologies). All samples showed common high quality RNA Integrity Numbers (RIN 9.0–9.6) and RNA was quantified by photometric measurement using a Nanodrop ND-1000 spectrophotometer (Thermo Scientific, Wilmington, DE, USA). A pool of amplified RNAs obtained from the Universal Mouse RNA (Stratagene, La Jolla, CA) was used as a reference. Briefly, 100 ng of total RNA was converted to cDNA, followed by in vitro transcription and incorporation of Cy5-dCTP (test) and Cy3-dCTP (reference) into nascent cRNA. After fragmentation, labeled cRNA was hybridized to Agilent SurePrint G3 Mouse GE 8x60K Microarrays for 17 h at 65 °C. Quality control parameters of cRNA labeling and hybridization performance were found within the manufacturers specifications. Arrays were scanned as described by the manufacturer. Signal intensities on 20 bit tiff images were calculated by Feature Extraction software (FE, Vers. 10.7.1.1; Agilent Technologies, Palo Alto, CA). Normalized signal values were then obtained by Cy5/Cy3 ratio computing and logarithmic base 2 transformations with the GeneSpring GX12 software (Agilent). Before statistical analysis, a new quality control was performed to filter out questionable and outlier expression values. Anyone normalized expression value across the samples in each experimental condition that was further away from the average, was ruled out considering it as an outlier.

### GO Ontology analysis

In this study, we analyzed only those genes related with apoptosis included in the mentioned cDNA microarray (see Additional file 1: Table S1). For this purpose, we selected genes that almost one of the GO Ontology identifiers associated in the Agilent microarray annotation data was included in a list of 387 GO-IDs related with apoptosis in the Gene Ontology database of the annotation data package “GO.db” (*GO.db: A set of annotation maps describing the entire Gene Ontology*. R package version 3.1.2. Carlson M. http://bioconductor.org). This list of apoptosis GO-IDs was created from the total number of GO identifiers registered in this database (38027), selecting those GO-Terms-Name (TERM) or GO-Term-Definition (DEF) included the words “apoptosis and/or apoptotic process” (Additional file 2: Table S2).

### Quantitative real-time reverse transcription polymerase chain reaction (RT-PCR) analysis

Total RNA was extracted using Trizol reagent (Invitrogen) according to the manufacturer’s instructions. Extracted RNAs were then quantified using a Nanodrop ND-1000 spectrophotometer (Thermo Scientific, Wilmington, DE, USA). Reverse transcription was performed using 1 μg of total RNA for cDNA synthesis with the High Capacity RNA-to-cDNA reverse transcription kit (Applied Biosystems, Foster City CA, USA) using random oligonucleotide primers. All the quantitative real-time PCR were carried out in 10 μl volume on ABI 7900HT Real-Time PCR system (Applied Biosystems, Carlsbad, CA) into 384-well plates using GoTaq qPCR Master Mix (Promega, Madison, WI). Amplicons were designed to span intron-exon boundaries. Primer efficiencies were calculated prior to experimental use and amplification efficiencies were greater than 90 % for all primer sets. Amplifications using specific primers (Additional file 3: Table S3) were done with a denaturation step at 95 °C for 2 min, followed by 40 cycles of denaturation at 95 °C for 3 s and primer annealing at 59 °C for 30 s. Upon completion of the cycling steps, a final step at 95 °C for 15 s, 60 °C for 15 s, and 95 °C for 15 s was done and then the reaction was stored at 4 °C. Reactions were run in triplicate in three independent experiments. The geometric mean of housekeeping genes HPRT1 and PPIA were used as an internal control to normalize the variability in expression levels and were analyzed using the 2^-ΔΔCT^ method described elsewhere [25].

### Western blot analysis

Isolated thymocytes were homogenized in radioimmunoprecipitation assay (RIPA) lysis buffer supplemented with PhosStop phosphatase inhibitor and Complete EDTA free protease inhibitors (Roche Molecular Biochemicals, Mannheim, Germany). The concentrations of thymocytes proteins were measured using a Pierce BCA Protein assay kit (ThermoFisher Scientific, Wilmington, DE) and a Benchmark Microplate Reader (Bio-Rad, Hercules, CA). Proteins (5 μg) were mixed in equal volume with 2× sample buffer (0.125 mol/l Tris-HCl, 4 % SDS, 20 % glycerol, 10 % 2-mercaptoethanol, 0.002 % bromophenol blue, pH 6.8). These samples were boiled at 99 °C for 5 min, and immediately cooled on ice. Electrophoresis was performed using 4–15 % Mini-PROTEAN TGX™ precast gels (BioRad, Hercules, CA). The proteins of electrophoresed gels were transferred to a polyvinylidene difluoride membrane (PVDF) (Millipore, Temecula*,* CA). The sizes of proteins were confirmed with the Precision Plus Protein Dual Color Standards (Bio-Rad).

After blocking, membranes were incubated with each primary antibody in either 5 % w/v *BSA* or nonfat dry milk, 1× TBS, 0.1 % Tween 20 overnight at 4 °C. As primary antibodies, p53 (1C12) (#2524; Cell Signaling), caspase-3 (Asp175) (#9661; Cell Signaling), and phospho-p38MAPK (Thr180/Tyr182) (#9211; Cell Signaling) were used at 1:1000 dilution. For β-actin (A 5316; Sigma) was used a dilution of 1:5000. Phospho-p53 (Ser15) (#9284; Cell Signaling) and phospho-p53 (Ser392) (#9281; Cell Signaling) were used at 1:500. Membranes were washed with TBST buffer and incubated for 60 min at room temperature with secondary antibodies we used Anti-rabbit IgG, HRP-linked Antibody (#7074; Cell Signaling) and Anti-mouse IgG, HRP-linked Antibody (#7076; Cell Signaling) at 1:1000 dilution. Bound antibodies were visualized by chemiluminescence using WesternBright™ ECL detection kit reagent (Advansta*,* Menlo Park*,* CA)*.* Luminescent images were analyzed using ImageQuant LAS 4000 biomolecular imager analyzer (GE Healhcare, Buckinghamshire, UK)*.* For densitometry analysis Scion Image software (Scion Corporation) analyzer program was used.

### Statistical analysis

#### cDNA microarray data analysis

To find sex differences in the transcriptional expression of genes involved in apoptosis signaling that are influenced by RAR, male and female transcriptional expression were compared under all experimental conditions by performing the statistical contrasts as follows: contrast 1 (male RAR vs female RAR); contrast 2 (male RAR - female RAR) vs (male control - female control); contrast 3(male RAR - female RAR) vs (male high dose irradiation - female high dose irradiation); and contrast 4 (male RAR - female RAR) vs (male low dose irradiation - female low dose irradiation). For those genes differentially expressed in all the contrasts, the difference between their expressions in male and female in RAR radiation condition is significant from any other difference found in any other radiation or control condition. These genes were selected as endpoints of this analysis.

The log-transformed (base 2) normalized values of expression from cDNA microarray experiments were used as the source of all raw data for statistical analysis in this section, and to detect differentially expressed genes in the contrasts described before, moderated *t*-test analysis was conducted with functions of the limma Package (R statistical software). *P* values were adjusted by the Benjamini-Hochberg method to control false discovery rate (FDR). And to consider a gene as differentially expressed, results from moderated t-test should show a FDR <0.05, and there should be an expression fold-change (FC) ≥1.5.

Additionally, to assess sex differences in gene expression taking into account at the same time the effect of the radiation treatment used, a two way ANOVA analysis was also applied.

#### Analysis of RT-PCR data

Mean expression values of selected genes were also calculated by quantitative real-time RT-PCR between RAR and the control condition in each sex, and compared using t-tests.

#### Analysis of protein expression data

To assess the sex differences across the experimental groups in protein amounts detected by Western blot analyses, multivariate linear regression models were performed including normalized mean values obtained from three independent experiments as the outcome, and sex, type of radiation treatment and an interaction term between them as independent variables.

#### Correlation coefficient analysis

Pearson product-moment correlation coefficients and their statistical significance were calculated to assess the strength and the direction of the associations between expressions.

All statistical analyses were performed using R Software (R Core Team (2013). R: A language and environment for statistical computing. R Foundation for Statistical Computing, Vienna, Austria. URL http://www.R-project.org/).

## Results

### Priming low dose radiation prior to high-dose radiation induced differential apoptotic rates in thymocytes of male and female mice

**Fig. 2:**
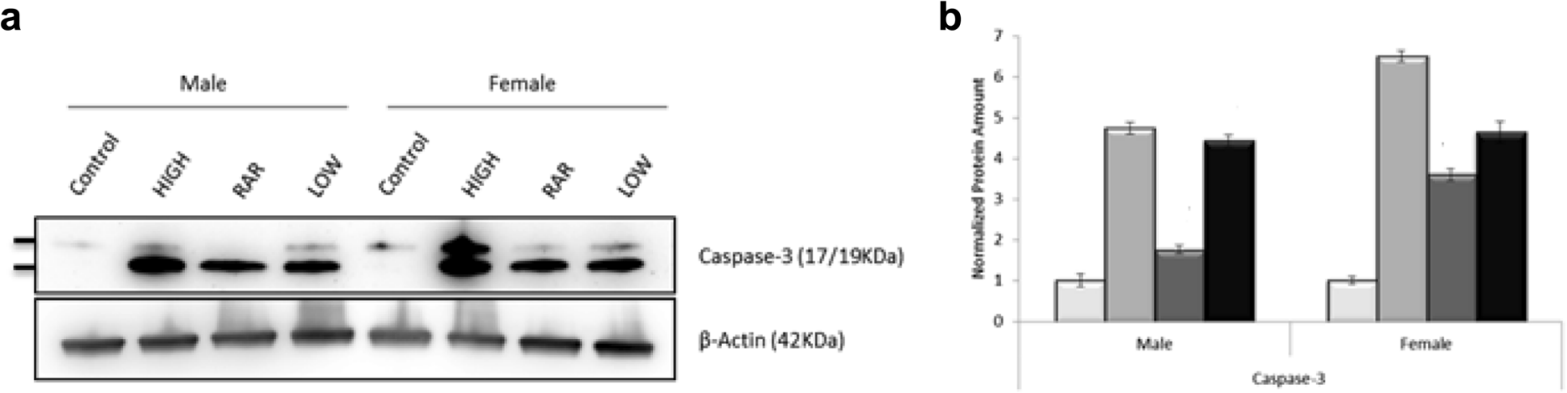
Dissimilar adaptive response of thymocyte apoptosis induced by priming low dose X-ray irradiation in C57BL/6 male and female mice. **a** Representative results of the Western blotting assay for the expression of caspase-3. *HIGH* indicates exposure to 1.75 Gy of γ-rays. RAR represents radio-adaptive response. *LOW* denotes exposure to 0.075 Gy of X-rays. **b** Quantitative analysis of caspase-3 protein levels. Amounts of caspase-3 were first normalized to β-actin, and then the ratio of each normalized value to its corresponding non-irradiated control value was calculated. Histograms represent the mean ± SD of normalized values obtained from three independent experiments. Open columns, non-irradiated thymocytes. *Light gray* columns, thymocytes exposed to 1.75 Gy of γ-rays. *Dark gray* columns, thymocytes treated with priming low dose X-irradiation (0.075 Gy) 6 h before a challenging high dose of γ-rays (1.75 Gy). *Black* columns, thymocytes exposed to 0.075 Gy of X-rays

We first analyzed the levels of apoptosis by measuring the amounts of caspase-3 protein in thymocytes of male and female C57BL/6 mice irradiated with an acute single dose of 0.075 Gy of X-rays or 1.75 Gy of γ-ray, and those treated with 0.075 Gy of priming X-rays followed in 6 h by 1.75 Gy of challenging γ-rays (hereafter referred as adaptive regimen of radiation) (Fig. 2). According to the multivariate regression model described in the Material and Methods section, we found significant differences in caspase-3 protein amounts for all radiation treatments relative to non-irradiated controls (Table 1). Moreover, there is an interaction between sex and treatment. In the particular case of RAR, the difference in the levels of caspase-3-induced apoptosis between radio-adapted thymocytes and non-irradiated thymocytes was higher in females as compared with males (Table 1).

**Table 1:** Multivariate analysis of caspase 3 protein expression

Gene	Variable	Value	Estimate	t value	*p* value	*p* value*
Caspase 3	Sex	Female	ref			1.010E-09
		Male	−6.559E-03	−0.043	0.966	
	Treatment	Control	ref			<2.2E-16
		High	5.502	36.221	<2E-16	
		RAR	2.592	17.062	1.090E-11	
		Low	3.651	24.040	5.520E-14	
	Sex*Treatment	Male(Control)*Female(Control)	ref			9.483E-08
		Male(High)*Female(High)	−1.752	−8.156	4.310E-07	
		Male(RAR)*Female(RAR)	−1.829	−8.513	2.450E-07	
		Male(Low)*Female(Low)	−0.221	−1.029	0.319	

Sex*Treatment interaction term between sex and radiation treatment, *ref* reference value, *Estimat e* regression coefficient, *t value* t test values; p value* p value from F test in ANOVA analysis, *High* exposure to 1.75 Gy of g-rays, *RAR* radio-adaptive response, *Low* exposure to 0.075 Gy of X-rays

### Microarray analysis identified apoptotic genes with differential expression in thymocytes of male and female mice exposed to the adaptive regimen of radiation

To explore sex-specific differences on global gene expression associated to RAR we carried out transcriptomic profiling in thymocytes of radioadapted males and females by analyzing cDNA probes from 1944 genes involved in apoptosis signaling (included in the Agilent Mouse Gene Expression G3 8x60K) according to GO-Ontology database. This analysis revealed that significant differential expression for 17 genes (log_2_FC >1.5, adjusted *p*-value (FDR) <0.05) in radio-adapted thymocytes of males as compared to females (Table 2). Moreover, according with the results of the two way ANOVA, the difference in the expression of these 17 genes between males and females depend on the radiation treatment used (Table 2).

**Table 2:** Genes involved in apoptosis signaling that show differential transcriptional expression between male and female radioadaptive thymocytes

Probe Name	Gene Symbol	Gene description	log_2_FC	FDR^a^	FDR^b^	GO.Ontology
A_51_P449995	*C6*	Complement component 6	−1.64	9.89E-03	2.75E-02	GO:0006919
A_51_P458451	*Adipoq*	Adiponectin	2.36	2.51E-03	3.76E-03	GO:0033034
A_51_P473170	*Tnfsf4*	Tumor necrosis factor superfamily member 4	2.06	1.67E-04	2.61E-03	GO:0043066
A_52_P304720	*Crlf1*	Cytokine receptor-like factor 1	−1.97	1.54E-03	4.64E-03	GO:0043524
A_52_P442710	*Ndnf*	Neuro-derived neurotrophic factor	2.18	2.20E-04	2.06E-03	GO:0043524
A_52_P464831	*Dlx1*	Distal-less homeobox 1	−2.45	1.50E-04	4.61E-03	GO:0043524
A_55_P1952533	*Fis1*	Fission 1 (Mitochondrial Outer Membrane) Homolog (*S. Cerevisiae*)	−2.94	1.06E-04	9.86E-04	GO:0001836
A_55_P1974019	*Dapk1*	Death associated protein kinase 1	2.18	9.38E-04	7.94E-03	GO:0008624
A_55_P1974845	*Pde1a*	Phosphodiesterase 1A, Calmodulin-Dependent	−2.24	2.46E-04	3.24E-03	GO:0034391
A_55_P1977431	*Cck*	Cholecystokinin	1.79	2.67E-04	2.45E-03	GO:0005044
A_55_P1982400	*Scara5*	Scavenger Receptor Class A, Member 5	−2.91	1.72E-04	3.91E-03	GO:0005044
A_55_P2042101	*Prune2*	Prune Homolog 2 (*Drosophila*)	−2.17	2.67E-04	6.68E-03	GO:0006917
A_55_P2044143	*Loxl4*	Lysyl Oxidase-Like 4	−2.29	4.37E-05	3.99E-04	GO:0005044
A_55_P2089710	*Ednrb*	Endothelin Receptor Type B	2.57	4.02E-04	1.69E-02	GO:0043066
A_55_P2144686	*Dmbt1*	Deleted In Malignant Brain Tumors 1	2.78	2.61E-05	2.46E-03	GO:0005044
A_55_P2268221	*Arhgap10*	Rho GTPase Activating Protein 10	−2.49	2.54E-04	3.04E-03	GO:0043066
A_55_P2280821	*Dlc1*	Deleted in liver cancer 1	−2.53	2.02E-05	4.49E-04	GO:0006919

*log*_*2*_ *FC* log_2_ Fold Change in gene expression between male and female under RAR treatment; a minus sign denotes down-regulation in radioadapted thymocytes of males as compared with females; *FDR* ^*a*^ FDR obtained from the moderated t-test for identifying differentially expressed genes between male and female RAR, *FDR* ^*b*^FDR obtained from two-way ANOVA for assessing gene expression differences between male and female regarless treatment

The 17 genes selected and shown in Table 2 were grouped in two clusters of differentially expressed genes. One composed by 10 genes that displayed a significant reduction in their transcript levels in males relative to females (including among others the pro-apoptotic genes *C6*, *Dlc1*, *Fis1*, *Loxl4*, *Prune2* and *Scara5*), whereas an inverse pattern was observed for the remaining seven genes (including the anti-apoptotic genes *Adipoq*, *Cck, Dmbt1*, *Ednrb*, *Ndnf* and *Tnfsf4*). These findings are consistent with the lesser levels of apoptosis observed in radio-adapted thymocytes of males as compared to those of females.

Notably, two of the 17 genes that showed sex-dependent transcriptional variations in radio-adapted thymocytes*, Fis1* and *Dlc1*, are closely related to *Trp53*-mediated apoptosis. Like *Trp53*, the *Fis1* gene participates in apoptosis induced by caspase 3 by acting directly at mitochondria [26]. Meanwhile, *Dlc1* is a transcription target of TRP53 [27] that is also involved in caspase-3-mediated apoptosis [28]. However, if we compare the levels of expression of these genes between RAR and the control situation, we found a significant differential expression of *Fis1* in both sexes with increased expression in females (log_2_FC = 1.97 and FDR = 0.0004) and reduced mRNA transcript levels in males (log_2_FC = −1.60, and FDR =0.001), while up-regulation of *Dlc1* expression was detected exclusively in the case of females (log_2_FC = 2.35, FDR = 0.00001). Quantitative real-time RT-PCR analyses (using primers showed in Additional file 3: Table S3) showed overexpression of *Fis1* (mean ± SD = 2.88 ± 0.19, *t* = 18.73, *P* <0.0001) and *Dlc1* (mean ± SD = 4.36 ± 0.26, *t* = 30.79, *P* <0.0001) in radio-adapted thymocytes of females compared to control samples, and down-modulation of *Fis1* (mean ± SD = 0.82 ± 0.06, *t* = −4.71, *P* = 0.003) in thymocytes of radio-adapted males relative to controls.

### Sex differences in the phosphorylation levels of TRP53 protein at serine 18 and serine 389 in radio-adapted thymocytes

**Fig. 3:**
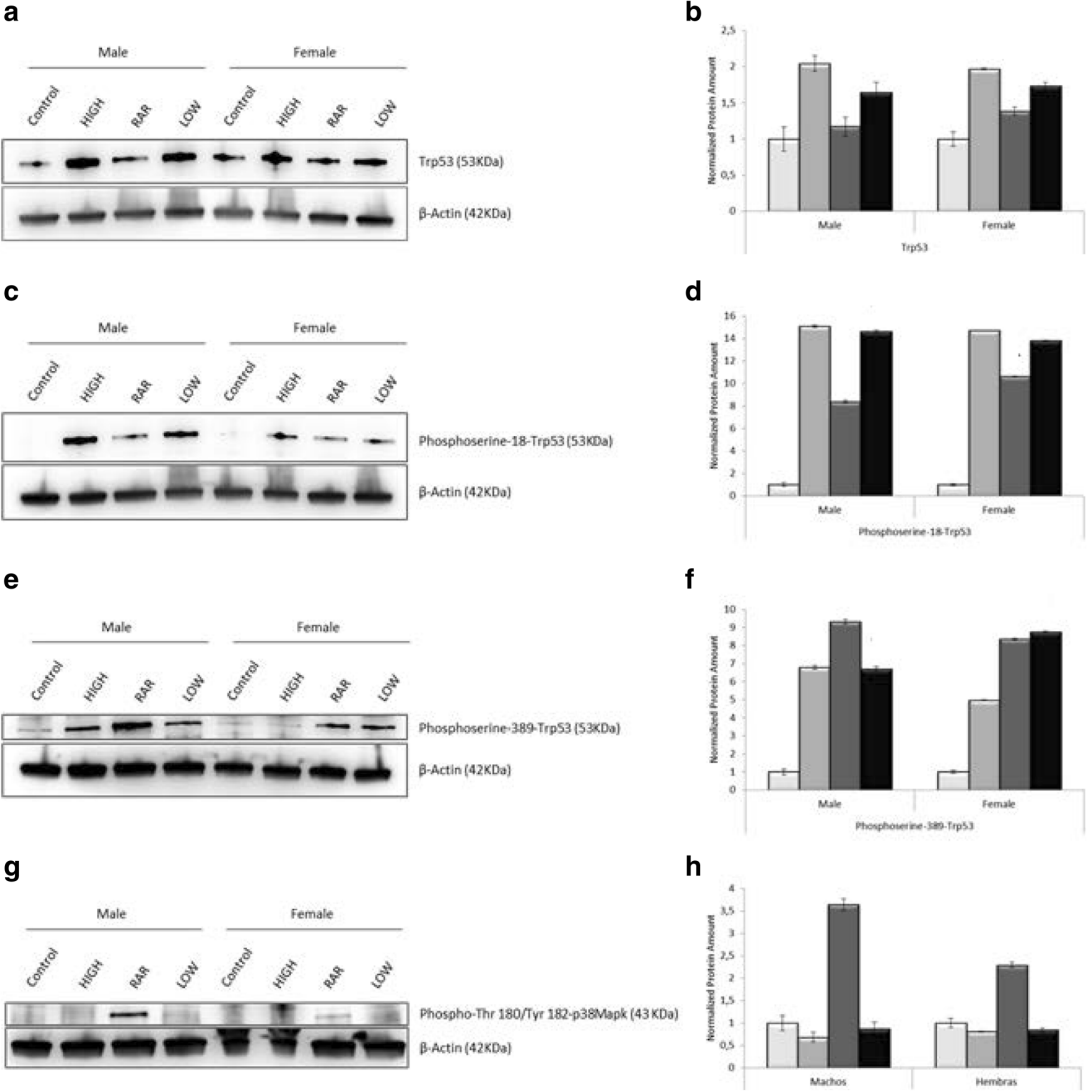
Different protein levels of phosphoserine-18-TRP53, phosphoserine-389-TRP53 and activated-p38MAPK after priming low dose X-ray irradiation in thymocytes of C57BL/6 male and female mice. *Left* panels, representative Western blots. *HIGH* indicates exposure to 1.75 Gy of γ-rays. RAR represents radio-adaptive response. *LOW* denotes exposure to 0.075 Gy of X-rays. *Right* panels, quantitative data showing normalized relative protein amounts. Each column represents mean of three independent experiments; *bars*, SD. Open histograms, non-treated thymocytes. *Light gray* histograms, thymocytes exposed to high dose (1.75 Gy) γ-irradiation. *Dark gray* histograms, thymocytes exposed to the adaptive regimen of radiation. *Black* histograms, thymocytes treated with low dose X-irradiation (0.075 Gy). **a**-**b**, TRP53. **c**-**d** Phosphorylated form of TRP53 at serine 18. **e**-**f** Phosphoserine-389-TRP53. **g**-**h** Phospho-(Thr180/Tyr182)-p38MAPK

Given that the predominant regulation of *Trp53* occurs at the post-translational level [29], it was not surprising that transcriptome analysis did not detect significant differences in the *Trp53* gene expression levels between radio-adapted thymocytes of males and females (Table 2). Thus, we analyzed TRP53 protein and its phosphorylated form at serine 18 under our experimental conditions. TRP53 protein expression in radio-adapted thymocytes was almost identical to that found in the control group (Fig. 3a-b). However, phosphoserine-18-TRP53 expression was strikingly increased in thymocytes exposed to the adaptive regimen of radiation (Fig. 3c-d) and the multivariate analysis showed significant sex differences in the expression of phosphoserine-18-TRP53 in radio-adapted thymocytes relative to controls, being these increases higher in females than in males (Table 3). Given that there seems to be a relationship between the levels of phosphorylation of TRP53 at serine 18 and the induction of apoptosis in response to ionizing radiation [30], we wanted to find out whether these two variables were correlated in radio-adapted thymocytes. Correlation analysis demonstrated that phosphoserine-18-TRP53 accumulation associated with increased amounts of caspase-3 protein in both males (*r* = 0.998, *t* = 21.0855, df = 1, *P* = 0.03) and females (*r* = 0.9995, *t* = 31.6768, df = 1, *P* = 0.02).

**Table 3:** Multivariate analysis of TRP53, phosphoserine-18-TRP53, phosphoserine-389-TRP53 and p38MAPK protein expression

Gene	Variable	Value	Estimate	t value	*p* value	*p* value*
TRP53	Sex	Female	ref			0.1805
		Male	−0.028	−0.305	0.764249	
	Treatment	Control	ref			1.033E-10
		High	1.02	11.110	6.23e-09	
		RAR	0.438	4.779	0.000205	
		Low	0.787	8.590	2.18e-07	
	Sex*Treatment	Male(Control)*Female(Control)	ref			0.2020
		Male(High)*Female(High)	0.104	0.800	0.435191	
		Male(RAR)*Female(RAR)	−0.183	−1.417	0.175527	
		Male(Low)*Female(Low)	−0.065	−0.501	0.623391	
phosphoserine-18-TRP53	Sex	Female	ref			0.0004246
		Male	−0.126	−0.979	0.3420	
	Treatment	Control	ref			<2.2E-16
		High	13.57	105.038	<2E-16	
		RAR	9.49091	73.469	<2E-16	
		Low	12.67	98.071	<2E-16	
	Sex*Treatment	Male(Control)*Female(Control)	ref			7.153E-11
		Male(High)*Female(High)	0.516	2.825	0.0122	
		Male(RAR)*Female(RAR)	−2.101	−11.498	3.81e-09	
		Male(Low)*Female(Low)	0.947	5.185	9.03E-05	
phosphoserine-389-TRP53	Sex	Female	ref			1.133E-05
		Male	0.058	0.854	0.406	
	Treatment	Control	ref			<2.2E-16
		High	4.041	59.194	<2E-16	
		RAR	7.419	108.665	<2E-16	
		Low	7.739	113.354	<2E-16	
	Sex*Treatment	Male(Control)*Female(Control)	ref			<2.2E-16
		Male(High)*Female(High)	1.748	18.102	4.43E-12	
		Male(RAR)*Female(RAR)	0.909	9.416	6.30E-08	
		Male(Low)*Female(Low)	−2.035	−21.076	4.26E-13	
phospho-Thr 180/Tyr 182-p38MAPK	Sex	Female	ref			1.196E-05
		Male	4.586E-16	0.000	1.0000	
	Treatment	Control	ref			1.368E-15
		High	−1.923e-01	−1.918	0.0732	
		RAR	1.291	12.874	7.38E-10	
		Low	−1.535e-01	−1.531	0.1453	
	Sex*Treatment	Male(Control)*Female(Control)	ref			3.259E-08
		Male(High)*Female(High)	−1.257e-01	−0.886	0.3886	
		Male(RAR)*Female(RAR)	1.348	9.507	5.53E-08	
		Male(Low)*Female(Low)	2.765E-02	0.195	0.8479	

Sex*Treatment interaction term between sex and radiation treatment, *ref* reference value, *Estimat e* regression coefficient, *t value* t test values, p value* p value from F test in ANOVA analysis, *High* exposure to 1.75 Gy of g-rays, *RAR* radio-adaptive response, *Low* exposure to 0.075 Gy of X-rays

In addition, highest increases in the phosphorylation levels of TRP53 at serine 389 were detected in thymocytes of mice exposed to adaptive regimen of radiation compared to non-irradiated thymocytes (Fig. 3e-f). Once more, we also observed gender differences in radio-adaptive thymocytes with males showing significant higher levels of phosphoserine-389-TRP53 expression than females (Table 3). Finally, we found an inverse relationship with a significant correlation between amounts of caspase-3 protein and levels of phosphoserine-389-TRP53 in radio-adapted thymocytes of males (*r = −*0.99993*,* t = −87.7847, df = 1, *P* = 0.007) and females (*r* = −0.9995*,* t *= −31.6768,* df = 1*, P* = 0.02)*.* These results suggest a protective effect against apoptosis for phosphoserine-389-TRP53 induced by RAR with a differential response between both sexes.

To confirm these latter results, we analyzed the phosphorylation levels of p38 mitogen activated protein kinase (p38MAPK) on threonine 180 and tyrosine 182 residues, since previous in vitro studies have demonstrated that such activated form of p38MAPK physically associates with TRP53 to directly phosphorylate serine 389 [31]. Expression of activated-p38MAPK protein was parallel to the amount of phosphoserine-389-TRP53, with significant highest levels of activated-p38Mapk detected in radio-adapted thymocytes of male mice as compared to those of females (Fig. 3g-h; Table 3). In fact, a significant high correlation was observed between levels of phosphoserine-389-TRP53 and activated-p38MAPK in radio-adapted thymocytes of males (*r =* 0.99976*,* t *=* 46.5361, df = 1*, P =* 0.013) and females (*r* = 0.99979, t = 49.2148*,* df *=1, P* = 0.012).

## Discussion

In this study, we provide the first evidence of a sex-dependent protective effect of administering a single priming low dose of X-rays against the detrimental effects of a subsequent high dose γ-irradiation exposure, manifested by a reduced level of caspase-3-mediated apoptosis in thymocytes. The fact that radio-adapted thymocytes of males and females display a differential effect against apoptosis suggests the existence of sex-specific genetic factors associated with protection from apoptosis. These differences may be related to the expression of the pro-apoptotic genes *Dlc1* [28] and *Fis1* [26], since we have observed transcriptional up-regulation of *Dlc1* and *Fis1* in thymocytes of female mice upon the adaptive regimen of radiation whereas significant down-regulation of *Fis1* was found in male radio-adapted thymocytes.

The TRP53 protein regulates the transcription of many different genes in response to a wide variety of stress signals, being *Dlc1* one of these *Trp53*-responsive genes [27]. Upon activation, TRP53 regulates the transcription of *Trp53*-responsive genes [32]. It has been demonstrated that TRP53-serine 15 phosphorylation plays a critical role in stimulating transactivation at *Trp53*-responsive promoters [33]. Furthermore, TRP53 upregulates human *DLC1* promoter activity in a dose-dependent manner [27]. Interestingly, we found higher levels of TRP53 phosphorylation at serine 18 in female radio-adapted thymocytes as compared to those of males. Therefore, different amounts of phosphoserine-18-TRP53 might explain, at least in part, variations in *Dlc1* expression that exists in both sexes. However, the exclusive overexpression observed in radio-adapted thymocytes of females suggests additional suggests additional explanations. Investigators have found that TRP53-mediated regulation of promoters may be influenced by the existence of non-functional TRP53-binding sites [34]. In this scenario, it is conceivably to speculate that differences in the number of functional TRP53 responsive elements in the *Dlc1* promoter of females and males might affect differentially to its expression.

It is known that the human DLC1 protein participates in a signaling cascade which cleaves the precursor caspase-3 into caspase-3, thereby allowing it to induce apoptosis [28]. On the other hand, radiation-induced apoptosis of mouse thymocytes, estimated by caspase-3 activation, was found to be entirely dependent of the *Trp53* gene, since it was absent in *Trp53* knockout mice [35]. Here, we have demonstrated that radio-adapted thymocytes of females show higher amounts of caspase-3 when compared with those of males. Altogether, our results support a role for *Dlc1* in the induction of TRP53-mediated apoptosis associated with RAR in female thymocytes.

In this study, we demonstrated that up-regulation of *Dlc1* in radio-adapted thymocytes of females was accompanied by overexpression of *Fis1*. Interestingly, thymocytes of male mice exposed to the adaptative regimen of radiation showed significant down-modulation of *Fis1*. It is known that down-regulation of *Fis1* powerfully inhibits cell death [26]. In contrast, over-expression of *Fis1* has been reported to induce apoptosis by a mechanism which involves mitochondrial fragmentation followed by the release of cytochrome c [36]. Thus, less induction of caspase-3-mediated apoptosis in male radio-adapted thymocytes might be influenced, at least in part, by down-modulation of *Fis1*. On the other hand, it has been reported that the mouse TRP53 protein rapidly accumulates at mitochondria of thymocytes undergoing γ-radiation-induced caspase-3-mediated apoptosis [35]. In the mitochondria, TRP53 directly induces permeabilization of the outer mitochondrial membrane by forming inhibitory complexes with the anti-apoptotic BCLXL and BCL2 proteins, resulting in cytochrome c release and in caspase-3 activation by the intrinsic death pathway [37]. Evidence derived from the analysis of human cells showed that serine 392 phosphorylation (serine 389 in murine TRP53) plays a critical role in the stability of TRP53 protein by inhibiting its nuclear export mechanism, but it is not essential in transactivation [38]. Thus, highest increases found in the amount of phosphorylated form of TRP53 at serine 389 in male radio-adapted thymocytes might be hindering the transport of TRP53 from nucleus to mitochondria. This fact together with down-modulation of *Fis1* would be causing an inhibitory effect on the release of cytochrome c from the mitochondria, thereby reducing the levels of caspase-3 and ultimately the induction of apoptosis in males exposed to the adaptive regimen of radiation. In an opposite way, overexpression de *Fis1* might cooperate with up-regulation of *Dlc1* to sustain a stronger caspase 3-mediated apoptotic response in radio-adapted thymocytes of females.

Finally, our results reveal that both activated-p38MAPK and phosphoserine-389-TRP53 are strongly correlated in RAR. It has been reported that the p38MAPK inhibitor, SB203850, blocked RAR but did not suppress apoptosis, indicating that the adaptive response and apoptosis are two complementary defense mechanisms via life-or-death decisions [12]. This study also demonstrated a pivotal role for TRP53 in channeling the radiation-induced double-strand breaks (DBSs) into an adaptive repair pathway, turning off the apoptotic signaling pathway. Our results support this notion since we report highest protein amounts of both p38MAPK and phosphoserine-389-TRP53 in male radio-adaptive thymocytes, which have in turn lowest levels of caspase-3-mediated apoptosis.

## Conclusions

We have observed a differential radio-adaptive response between male and female mice using as endpoint the induction of apoptosis mediated by caspase-3 in thymocytes. Radio-adaptive response in females was accompanied by higher protein amounts of phosphoserine-18-TRP53 and caspase-3 as compared with male RAR. We also found that the pro-apoptotic *Dlc1* and *Fis1* genes are specifically overexpressed in thymocytes of female mice exposed to the adaptive regimen of radiation. In contrast, a protective role against caspase-3-mediated apoptosis associated with an increase in the phosphorylation levels of TRP53 at serine 389 and down-modulation of *Fis1* was demonstrated in radio-adaptive thymocytes of male mice.

## Additional files

Additional file 1: Listing of genes involved in apoptosis included in the Agilent Mouse Gene Expression G3 8x60K microarray. (XLSX 420 kb) Additional file 2: Listing of GO-IDs related with apoptosis in the Gene Ontology database. (XLSX 52 kb) Additional file 3: Listing of primers used in the quantitative real-time RT-PCR analysis. (XLSX 10 kb)

## Abbreviations

FC: Fold-change
FDR: False discovery rate
IR: Ionizing radiation
RAR: Radio-adaptive response
RT-PCR: Reverse transcription polymerase chain reaction
